# Assessing Generative Pretrained Transformers (GPT) in Clinical Decision-Making: Comparative Analysis of GPT-3.5 and GPT-4

**DOI:** 10.2196/54571

**Published:** 2024-06-27

**Authors:** Adi Lahat, Kassem Sharif, Narmin Zoabi, Yonatan Shneor Patt, Yousra Sharif, Lior Fisher, Uria Shani, Mohamad Arow, Roni Levin, Eyal Klang

**Affiliations:** 1 Department of Gastroenterology Chaim Sheba Medical Center Affiliated with Tel Aviv University Ramat Gan Israel; 2 Department of Gastroenterology Samson Assuta Ashdod Medical Center Affiliated with Ben Gurion University of the Negev Be'er Sheva Israel; 3 Department of Internal Medicine B Sheba Medical Centre Tel Aviv Israel; 4 Department of Internal Medicine C Hadassah Medical Center Jerusalem Israel; 5 Division of Data-Driven and Digital Medicine (D3M) Icahn School of Medicine at Mount Sinai New York, NY United States

**Keywords:** ChatGPT, chat-GPT, chatbot, chatbots, chat-bot, chat-bots, natural language processing, NLP, artificial intelligence, AI, machine learning, ML, algorithm, algorithms, predictive model, predictive models, predictive analytics, predictive system, practical model, practical models, internal medicine, ethics, ethical, ethical dilemma, ethical dilemmas, bioethics, emergency medicine, EM medicine, ED physician, emergency physician, emergency doctor

## Abstract

**Background:**

Artificial intelligence, particularly chatbot systems, is becoming an instrumental tool in health care, aiding clinical decision-making and patient engagement.

**Objective:**

This study aims to analyze the performance of ChatGPT-3.5 and ChatGPT-4 in addressing complex clinical and ethical dilemmas, and to illustrate their potential role in health care decision-making while comparing seniors’ and residents’ ratings, and specific question types.

**Methods:**

A total of 4 specialized physicians formulated 176 real-world clinical questions. A total of 8 senior physicians and residents assessed responses from GPT-3.5 and GPT-4 on a 1-5 scale across 5 categories: accuracy, relevance, clarity, utility, and comprehensiveness. Evaluations were conducted within internal medicine, emergency medicine, and ethics. Comparisons were made globally, between seniors and residents, and across classifications.

**Results:**

Both GPT models received high mean scores (4.4, SD 0.8 for GPT-4 and 4.1, SD 1.0 for GPT-3.5). GPT-4 outperformed GPT-3.5 across all rating dimensions, with seniors consistently rating responses higher than residents for both models. Specifically, seniors rated GPT-4 as more beneficial and complete (mean 4.6 vs 4.0 and 4.6 vs 4.1, respectively; *P*<.001), and GPT-3.5 similarly (mean 4.1 vs 3.7 and 3.9 vs 3.5, respectively; *P*<.001). Ethical queries received the highest ratings for both models, with mean scores reflecting consistency across accuracy and completeness criteria. Distinctions among question types were significant, particularly for the GPT-4 mean scores in completeness across emergency, internal, and ethical questions (4.2, SD 1.0; 4.3, SD 0.8; and 4.5, SD 0.7, respectively; *P*<.001), and for GPT-3.5’s accuracy, beneficial, and completeness dimensions.

**Conclusions:**

ChatGPT’s potential to assist physicians with medical issues is promising, with prospects to enhance diagnostics, treatments, and ethics. While integration into clinical workflows may be valuable, it must complement, not replace, human expertise. Continued research is essential to ensure safe and effective implementation in clinical environments.

## Introduction

Artificial intelligence (AI), particularly chatbot systems, is becoming an instrumental tool in health care, aiding clinical decision-making and patient engagement [1,2]. These systems, using natural language processing (NLP), offer support to physicians in various clinical scenarios [3]. OpenAI’s GPT technology represents a leading example of this innovation [4].

The integration potential of AI in health care is not only a technological advancement; it is a paradigm shift in how medical professionals approach patient care. Leveraging AI, clinicians can access real-time data analysis, personalized treatment recommendations, and even predictive insights into patient health trends. The convergence of AI with traditional medical practices is fostering a new era of precision medicine, where treatments are tailored to individual patient needs and preferences. This personalized approach enhances patient satisfaction and outcomes, while also optimizing resource allocation within the health care system.

Earlier GPT models, like GPT-3.5, were recognized for generating human-like text and have potential across various fields, including medicine, where they can assist in diagnostics, therapeutic planning, telehealth, and patient education [5-15]. Recent AI algorithm’s ability to synthesize vast information and recognize complex patterns can enhance clinical accuracy, and reduce errors [16].

With the introduction of GPT-4, an advancement in AI’s capabilities, it becomes imperative to evaluate its comparative effectiveness in clinical scenarios [17]. This model, with improved language comprehension and generation, presents a promising opportunity for refined health care applications. Past studies have highlighted the clinical potential of ChatGPT (GPT-3.5), which managed to score approximately 60% on the USMLE (United States Medical Licensing Examination) [18]. Moreover, the more advanced GPT-4 achieved an impressive 87% [19].

Recent advancements in AI technology have paved the way for innovative applications in medical education and patient support, particularly through AI-powered chatbots. Chow et al [20] developed a chatbot using the IBM Watson Assistant platform to make radiotherapy knowledge more accessible to the general public. This chatbot, designed with a user-friendly interface, engages users in a digital question-and-answer format, enhancing their understanding of radiotherapy. Despite its positive feedback, the study highlights the need for improvements in conversational approaches and language inclusivity. In another study, Rebelo et al [21] created a novel web-based assistant to explain the radiation treatment process comprehensively. Using IBM Watson’s NLP features, this chatbot guides users through the complex radiotherapy process, from diagnosis to treatment delivery, and has been tested for effectiveness in knowledge transfer. Both studies underscore the potential of AI chatbots in transforming medical communication and education, offering personalized, digital, and reassuring platforms for patients and the general public to access critical health information. These initiatives reflect the growing importance of AI in addressing the need for accessible and accurate medical information, particularly in areas, like radiotherapy, where patient and family education is crucial.

However, the reliability of ChatGPT in delivering health information to patients and health care providers remains questionable. ChatGPT differs from specialized medical chatbots as it is not trained on data sets curated by medical professionals, raising concerns about the accuracy of its medical advice and ethical issues related to patient safety and data privacy. As ChatGPT evolves as a disruptive technology in health care, it faces several challenges. These include its database being possibly outdated, risks of misinformation, and the need for its integration into the medical field to be carefully monitored and guided by ethical frameworks and professional standards [22].

In light of all of the above, the goal of this study is to analyze the performance of ChatGPT-3.5 and ChatGPT-4 in addressing complex clinical and ethical dilemmas and to illustrate their potential role as allies in health care decision-making.

Our study aims to compare the free version of GPT-3.5 with the paid version of GPT-4 in a medical context, focusing on accessibility and performance for a diverse audience. This comparison is vital for understanding the practicality and effectiveness of AI in health care, especially for users with limited resources. By evaluating both versions, we can assess the performance gap, guide resource allocation, and address the democratization of AI technology in medicine. This approach not only helps in setting realistic expectations for the free version’s capabilities but also underscores the ethical and social implications of AI accessibility. Such a comparison is crucial for informing both users and developers about the practical use of AI in medical contexts, ensuring the study remains relevant and beneficial to a wide range of potential users including those without access to the paid version.

Furthermore, we aimed to assess and compare the performance of ChatGPT in responding to medical questions across different categories, namely emergency medicine, internal medicine, and ethical dilemmas. We specifically focus on comparing the ratings provided by 2 distinct groups: medical residents and senior physicians. The rationale behind this comparison lies in the differing levels of clinical experience and expertise between these groups. Medical residents, being in the earlier stages of their training, may approach medical questions with a fresh perspective and rely more on foundational knowledge and guidelines. On the other hand, senior physicians, with their extensive clinical experience, may offer nuanced insights and consider broader contextual factors in their assessments. By evaluating responses from both groups, we aimed to gain a comprehensive understanding of ChatGPT’s performance across various medical domains and discern potential differences in the depth and accuracy of responses based on clinical experience levels. We believe that this comparison can provide valuable insights into the utility and limitations of using AI language models, like ChatGPT, in supporting medical decision-making across different stages of clinical training and practice, and to contribute to a broader understanding of AI’s expanding role in the medical field.

## Methods

### Study Design

In this study, 3 forms of comparisons were conducted to evaluate the performance of ChatGPT in responding to medical questions. First, we compared the responses generated by 2 versions of the model, GPT-3.5 and GPT-4, to assess any differences in the quality and accuracy of responses between the 2 iterations. This comparison aimed to elucidate potential improvements or variations in performance introduced by advancements in the underlying AI architecture. Second, we compared the evaluations provided by senior physicians and medical residents on the responses generated by ChatGPT. This analysis aimed to explore potential disparities in the perceived quality and usefulness of responses based on the level of clinical experience and expertise of the evaluator. Finally, we analyzed responses across different types of medical questions, including emergency medicine, internal medicine, and ethical dilemmas, to assess the model’s performance across diverse clinical scenarios. This comparison aimed to identify any variations in the model’s ability to provide relevant and accurate information across various medical domains. By conducting these 3 forms of comparisons, we aimed to comprehensively evaluate the capabilities of ChatGPT in addressing medical queries across different contexts and user perspectives.

### Question Development and Categorization

A total of 4 experienced physicians specialized in internal medicine and emergency medicine collaborated to formulate 176 questions. Questions were generated based on clinical experience and designed to follow clinical guidelines.

The questions were written by 2 senior physicians in internal medicine and emergency medicine with reference to board questions used for the assessment of doctors in training, questions were chosen to reflect different aspects of medicine as seen in everyday practice. Verification was done independently by 2 different physicians and answers were checked to comply with updated guidelines.

Questions were mainly classified into one of three categories, (1) emergency room (ER): questions addressing emergency scenarios; (2) internal medicine: questions concerning diagnosis, treatment, and management of internal medicine conditions; and (3) ethical: questions focusing on ethical considerations in medical practice.

These questions were created to reflect real-world clinical scenarios, aligning with prevailing clinical guidelines and ethical norms.

### Selection and Configuration of Models

The study engaged OpenAI’s GPT-3.5 and GPT-4 models, recognized for their advanced capabilities in NLP. They were queried through OpenAI’s Application Programming Interface to generate answers to the formulated questions.

### Prompting the Models

The models were prompted using the specific instruction: “Please answer this {question_type} question in clear, concise, concrete, full, bullets: {question}.”

### Grading Participants and Process

A total of 8 clinicians from 2 tertiary medical centers, including 4 senior physicians and 4 residents, independently graded the responses. Senior physicians were all active in daily clinical practice, including emergency care within the ER department, between 2 and 4 years subsequent to the completion of residency training. All residents were in their last year of residency (3 out of 4 years) and experienced with internal and emergency medicine. The grading criteria are provided in Textbox 1.

A comprehensive grading guide ensured uniformity in the application of the criteria.

**Grading criteria.**

**Accuracy**

Does it reflect medical understanding (scale 1-5). The answers were graded as follows:

- Very inaccurate: the response shows a fundamental misunderstanding of medical concepts.
- Somewhat inaccurate: the answer contains more incorrect than correct medical information.
- Moderately accurate: the response is generally correct but includes some inaccuracies.
- Mostly accurate: the answer is largely accurate with minor errors or omissions.
- Completely accurate: the response reflects a high level of medical understanding with accurate and precise information.

**Relevance**

Does the response directly address the asked question, or does it deviate to unrelated subtopics (scale 1-5). The answers were graded as follows:

- Not relevant: the response is completely off-topic or unrelated to the question.
- Slightly relevant: the answer addresses the question but includes significant unrelated information.
- Moderately relevant: the response is relevant but includes some tangential content.
- Highly relevant: the answer is directly related to the question with minimal unrelated details.
- Completely relevant: the response precisely addresses the question without any deviation.

**Clarity**

How clear is the provided information (scale 1-5). The answers were graded as follows:

- Very unclear: the response is confusing, poorly articulated, or difficult to comprehend.
- Somewhat clear: the answer has some clarity but may require additional explanation.
- Clear: the response is understandable with a reasonable level of clarity.
- Very clear: the answer is well-explained and easy to follow.
- Exceptionally clear: the response is articulated in an exceptionally straightforward and comprehensible manner.

**Beneficial**

Does the response significantly aid the decision-making process (scale 1-5). The answers were graded as follows:

- Not beneficial: the response provides no useful aid for decision-making.
- Slightly beneficial: the answer offers limited assistance in the decision-making process.
- Moderately beneficial: the response is somewhat helpful for decision-making.
- Highly beneficial: the answer significantly aids the decision-making process.
- Extremely beneficial: the response is exceptionally valuable and decisively aids in making informed decisions.

**Completeness**

Does the response cover all necessary information required to fully answer the question (scale 1-5). The answers were graded as follows:

- Very incomplete: the response leaves out crucial information necessary to answer the question.
- Somewhat complete: the answer includes some necessary information but is missing significant aspects.
- Moderately complete: the response covers a fair amount of the necessary information.
- Mostly complete: the answer is almost complete with only minor omissions.
- Fully complete: the response comprehensively covers all information required to fully answer the question.

### Statistical Analysis

To evaluate the performance differences between GPT-3.5 and GPT-4, the nonparametric Mann-Whitney *U* tests were used. The Mann-Whitney *U* test was chosen to compare GPT-3.5 and GPT-4 scores, as our data are ordinal. This nonparametric test is ideal for data not meeting normal distribution assumptions. This nonparametric test was also used to discern grading disparities between senior physicians and residents, representing 2 heterogeneous grader groups with potentially varying evaluation patterns. Furthermore, to probe into the potential differences in the responses based on the 3 distinct question categories (emergency medicine, internal medicine, and ethical questions), Mann-Whitney *U* tests were once again applied.

For all statistical tests, a *P* value of less than .05 was considered indicative of a statistically significant difference.

## Results

### Overview of ChatGPT Performance

An evaluation was conducted to assess the performance of 2 generative models, GPT-4 and GPT-3.5, in terms of accuracy, relevance, clarity, benefit to the decision-making process, and completeness of the response. A total of 176 questions were analyzed by both residents and seniors, yielding a consistent and objective assessment. Examples of emergency medicine, internal medicine, and ethical questions and the corresponding GPT-3.5 and GPT-4 detailed answers are presented in Table 1.

GPT-4 demonstrated statistically significant superiority in all assessed criteria compared with GPT-3.5. The analysis shows that the models differed notably in their ability to provide clear, relevant, and beneficial responses, with GPT-4 consistently outperforming GPT-3.5. The results of GPT-4 and GPT-3.5 are summarized in Table 2.

**Table 1:** A comparison between the responses of ChatGPT-4 and ChatGPT-3.5 to questions from 3 different disciplines: emergency medicine, internal medicine, and ethical dilemmas.

Discipline	Question	ChatGPT-4	ChatGPT-3.5
Emergency medicine	How would you manage a patient presenting with signs of necrotizing fasciitis?	Immediately initiate broad-spectrum intravenous antibiotics including coverage for gram-positive, gram-negative, and anaerobic bacteria Administer intravenous fluid resuscitation to maintain hemodynamic stability Provide the patient with adequate analgesia for pain management Consult a surgeon urgently for possible surgical debridement. Necrotizing fasciitis is a surgical emergency and tissue debridement is critical in the management Order laboratory tests including complete blood count, electrolytes, renal function, liver function, coagulation profile, and blood cultures Obtain imaging, such as x-ray, CT^a^ scan, or magnetic resonance imaging, if the diagnosis is uncertain. However, do not delay treatment for imaging Consider administration of IVIG^b^ as adjunctive therapy. Monitor the patient closely in an intensive care setting Provide supportive care including nutritional support and prevention of secondary infections Consider tetanus prophylaxis if the patient’s immunization status is unknown or not up-to-date Arrange for psychosocial support as necrotizing fasciitis can have a significant impact on mental health Implement contact precautions to prevent the spread of infection to other patients and health care workers	Call for emergency medical assistance immediately Begin supportive care including oxygenation and intravenous fluids Administer broad-spectrum antibiotics that cover both gram-positive and gram-negative bacteria Obtain blood cultures and wound cultures Surgical consultation should be obtained immediately Consider early surgical intervention including debridement of affected tissue Monitor the patient closely for signs of sepsis and organ dysfunction Administer pain medication as needed Educate the patient and family about the disease and potential complications
Internal medicine	How should you handle a patient with known cirrhosis presenting with new-onset ascites?	Confirm the diagnosis: ascertain the patient’s symptoms such as abdominal bloating, weight gain, and discomfort. Perform a physical examination to assess for fluid waves or shifting dullness Order diagnostic tests: arrange for an ultrasound or CT scan to confirm ascites. A paracentesis may be performed to analyze the ascitic fluid and rule out infection or malignancy Manage underlying cirrhosis: ensure the patient is on optimal therapy for cirrhosis, which may include medications like diuretics, beta-blockers, or lactulose Sodium restriction: advise the patient to limit their sodium intake to less than 2 grams per day Diuretics: start the patient on a diuretic regimen, often spironolactone with or without furosemide, to help mobilize the fluid Monitor for complications: regularly monitor the patient for complications of cirrhosis and ascites such as hepatic encephalopathy, spontaneous bacterial peritonitis, and hepatorenal syndrome Consider referral for liver transplantation: if the patient’s condition continues to worsen despite optimal medical therapy, consider referral to a transplant center for evaluation for liver transplantation Patient education: educate the patient about the importance of medication adherence, dietary sodium restriction, and regular follow-up, and also, inform about the signs of complications	Obtain a detailed medical history, including the duration and severity of ascites, any recent changes in medication or diet, and any history of alcohol or drug abuse Perform a thorough physical examination including a careful assessment of the abdomen for fluid accumulation, signs of liver disease, and any evidence of hepatic encephalopathy Order laboratory tests including liver function tests, complete blood count, electrolytes, and renal function tests Monitor the patient closely for signs of complications, such as infection, renal failure, or hepatic encephalopathy, and adjust treatment as needed Educate the patient about the importance of adhering to a low-sodium diet, avoiding alcohol and other hepatotoxic substances, and following up regularly with their health care provider to monitor their condition.
Ethical questions	Should a health care provider give placebos to patients who insist on receiving treatment when no treatment is necessary?	Ethically, health care providers should not give placebos to patients who insist on receiving treatment when no treatment is necessary The use of placebos without patient consent is deceptive and violates the principles of patient autonomy and informed consent, which are fundamental to medical ethics It can undermine the trust between the patient and the health care provider. If discovered, it could damage the patient’s confidence in their health care provider or the health care system as a whole It may also delay necessary treatment if the patient’s condition changes or worsens, as the placebo effect is temporary and does not treat underlying conditions However, some argue that if a placebo can alleviate patient anxiety or perceived symptoms, it may be considered in the patient’s best interest. This approach should only be taken with full transparency and patient consent In conclusion, it is generally considered unethical to administer placebos without patient knowledge or consent, and it is crucial to respect patient autonomy, promote open communication, and provide appropriate care based on medical necessity	No, health care providers should not give placebos to patients who insist on receiving treatment when no treatment is necessary Giving placebos is unethical and goes against the principle of informed consent. It can also harm the patient’s trust in health care providers and the medical profession as a whole Health care providers should educate patients on the lack of necessity for treatment and offer alternative forms of support such as counseling or therapy If a patient continues to insist on treatment, it is important to have an open and honest conversation about the potential risks and benefits of treatment and the lack of evidence supporting the use of placebos

^a^CT: computed tomography.

^b^IVIG: intravenous immunoglobulin.

**Table 2:** Comparative analysis of GPT-4 and GPT-3.5 performance.

Criteria	GPT-4, mean (SD)	GPT-3.5, mean (SD)	*P* value
Accuracy	4.5 (0.8)	4.2 (0.9)	<.001
Relevance	4.5 (0.7)	4.4 (0.8)	<.001
Clarity	4.6 (0.7)	4.4 (0.9)	<.001
Beneficial	4.3 (0.9)	3.9 (1.0)	<.001
Completeness	4.3 (0.9)	3.7 (1.1)	<.001
Total	4.4 (0.8)	4.1 (1.0)	<.001

### Distribution of Ratings for GPT-4 and GPT-3.5

GPT-4 consistently scored between 4.29 and 4.55, excelling in clarity with an average of 4.55, indicating clear and direct information delivery. In contrast, GPT-3.5 scores ranged from 3.92 to 4.37, with its lowest in beneficiality at 3.92, reflecting variability in aiding decision-making.

For all the rating dimensions, GPT-4 generally received higher ratings than its counterpart, GPT-3.5. The mode—the most frequently given rating—for GPT-4 consistently achieved the maximum score of 5 across all categories, except for the benefit category, where the mode was approximately 4.5.

In contrast, the distribution of ratings for GPT-3.5 presented a wider spread, with modes fluctuating between scores of 3 and 5, contingent on the category. In particular, the categories of benefit and completeness exhibited a broad spread of ratings for GPT-3.5, indicative of greater variability in the responses.

### Comparison Between Residents and Seniors

In evaluating the models’ responses based on the reviewer type (Table 3), significant differences were discerned between residents and senior physicians.

**Table 3:** Comparison between residents’ and seniors’ assessments of GPT-4 and GPT-3.5 answers.

Criteria	Residents GPT-4, mean (SD)	Seniors GPT-4, mean (SD)	*P* value GPT-4	Residents GPT-3.5, mean (SD)	Seniors GPT-3.5, mean (SD)	*P* value GPT-3.5
Accuracy	4.3 (0.9)	4.7 (0.5)	<.001	4.1 (1.0)	4.3 (0.7)	<.001
Relevance	4.3 (0.8)	4.7 (0.6)	<.001	4.2 (0.9)	4.6 (0.8)	<.001
Clarity	4.4 (0.7)	4.7 (0.6)	<.001	4.2 (0.9)	4.5 (0.9)	<.001
Beneficial	4.0 (1.0)	4.6 (0.7)	<.001	3.7 (1.0)	4.1 (1.0)	<.001
Completeness	4.1 (1.0)	4.6 (0.6)	<.001	3.6 (1.2)	3.9 (0.9)	<.001

For both GPT-4 and GPT-3.5, seniors consistently rated the responses higher across all criteria. The variation was most notable in the assessment of the model’s benefits and completeness.

In the case of GPT-4, senior physicians rated it as more beneficial (4.6 vs 4.0, *P*<.001) and more complete (4.6 vs 4.1, *P*<.001) compared with the residents. A similar trend was observed for GPT-3.5, with seniors appreciating its benefits (4.1 vs 3.7, *P*<.001) and completeness (3.9 vs 3.5, *P*<.001) more than the residents. This finding suggests a potential influence of clinical experience on the perception of AI-generated responses.

### Performance Across Question Types

**Figure 1:**
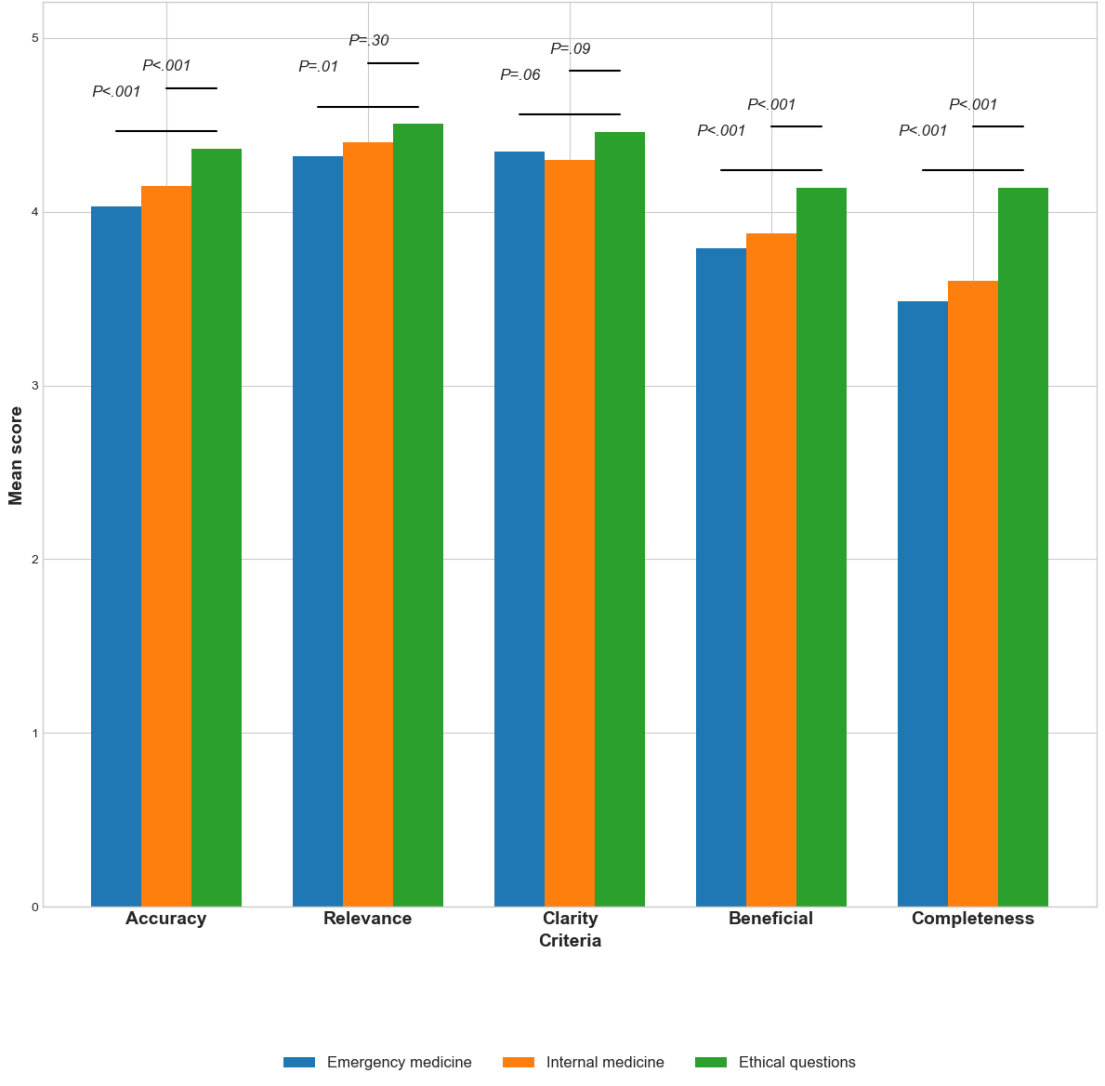
Performance of GPT-3.5 according to question subject.

**Figure 2:**
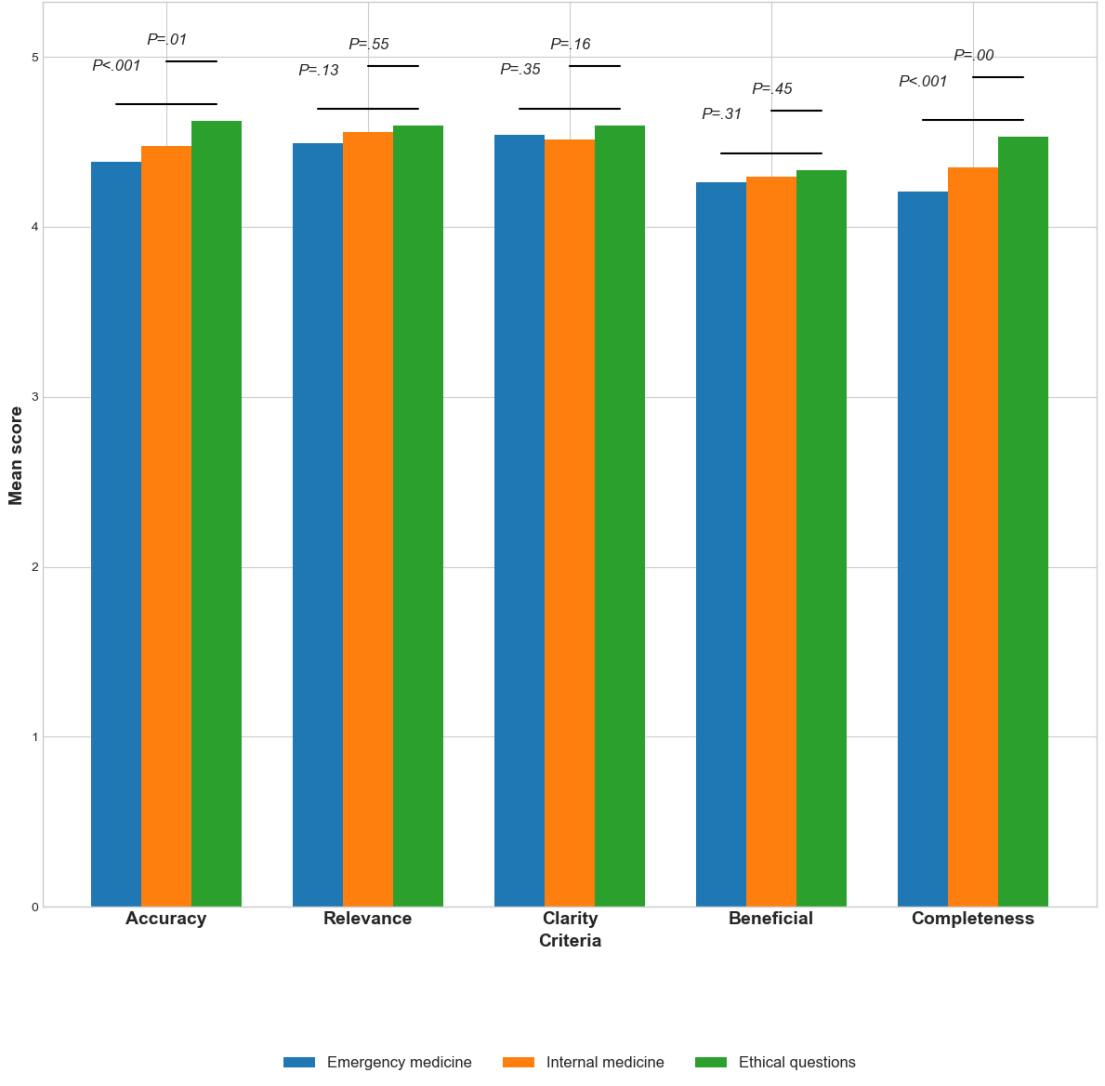
Performance of GPT-4 according to question subject.

In the evaluation of both GPT-4 and GPT-3.5, distinctions were evident across question types (emergency medicine, internal medicine, and ethical questions). Ethical queries consistently received the highest ratings (Figures 1 and 2 present analysis for GPT-4 and GPT-3.5 across all reviewers, while Tables 4 and 5 present analysis separately for residents and seniors). Higher grades were given for ethical answers by residents and seniors alike and in both models of GPT (Tables 4 and 5).

**Table 4:** Comparison between residents’ and seniors’ assessment of medical and ethical GPT-4 answers.

Criteria		Medical GPT-4, mean (SD)	Ethical GPT-4, mean (SD)	*P* value
**Residents’ assessment**				
	Accuracy	4.2 (0.9)	4.4 (0.8)	.17
	Relevance	4.3 (0.8)	4.4 (0.7)	.52
	Clarity	4.4 (0.7)	4.5 (0.7)	.17
	Beneficial	3.9 (1.0)	4.1 (0.9)	.03
	Completeness	4.0 (1.0)	4.3 (0.8)	<.001
	Total	4.2 (0.9)	4.3 (0.8)	<.001
**Seniors’ assessment**				
	Accuracy	4.6 (0.6)	4.9 (0.3)	<.001
	Relevance	4.7 (0.6)	4.8 (0.5)	.14
	Clarity	4.7 (0.7)	4.7 (0.6)	.85
	Beneficial	4.6 (0.7)	4.5 (0.8)	.63
	Completeness	4.5 (0.7)	4.8 (0.5)	<.001
	Total	4.6 (0.7)	4.7 (0.6)	<.001

**Table 5:** Comparison between residents’ and seniors’ assessment of medical and ethical GPT-3.5 answers.

Criteria		Medical GPT-3.5, mean (SD)	Ethical GPT-3.5, mean (SD)	*P* value
**Residents’ assessment**				
	Accuracy	4.0 (1.0)	4.2 (0.9)	.08
	Relevance	4.1 (0.9)	4.3 (0.8)	.004
	Clarity	4.2 (0.9)	4.3 (0.8)	.11
	Beneficial	3.6 (1.0)	3.9 (1.0)	<.001
	Completeness	3.4 (1.2)	3.9 (1.0)	<.001
	Total	3.9 (1.0)	4.1 (0.9)	<.001
**Seniors’ assessment**				
	Accuracy	4.1 (0.8)	4.6 (0.5)	<.001
	Relevance	4.6 (0.9)	4.7 (0.6)	.70
	Clarity	4.5 (1.0)	4.6 (0.8)	.16
	Beneficial	4.0 (1.0)	4.4 (1.0)	<.001
	Completeness	3.7 (0.9)	4.3 (0.7)	<.001
	Total	4.2 (1.0)	4.5 (0.8)	<.001

For GPT-4, mean scores were fairly consistent across the criteria of accuracy, and completeness, with significant differences among the question types (Figure 1). Specifically, completeness revealed statistically significant differences among emergency, internal, and ethical questions (4.2, SD 1.0; 4.3, SD 0.8; and 4.5, SD 0.7; *P*<.001).

For GPT-3.5, differences among question types were often statistically significant, as demonstrated in Figure 2. Accuracy ranged from 4.0 (SD 0.9) for ED questions to 4.4 (SD 0.8) for ethical questions (*P*<.001). Similar trends were observed in beneficial (*P*<.001) and completeness (*P*<.001).

## Discussion

### Principal Findings

In our study, we evaluated ChatGPT-3.5 and ChatGPT-4’s ability to address complex clinical and ethical dilemmas. Both models showed promise as tools to aid physicians in decision-making. Notably, ChatGPT-4 outperformed ChatGPT-3.5 across all parameters. This was validated by both resident and senior medical practitioners.

In an effort to provide a thorough, wide-ranging, and accurate evaluation, our study included an extensive compilation of queries, totaling 176 questions. These questions spanned various topics found within the domains of emergency medicine, internal medicine, and ethical considerations, mirroring the real-life challenges that doctors frequently face in their daily clinical work. Both groups of evaluators—senior physicians and interns—assessed each question, aiming to capture diverse perspectives on the perceived benefits at different levels of expertise. To the best of our knowledge, this is the most exhaustive study conducted on this particular subject.

Overall, both ChatGPT models received high grades in terms of accuracy, relevance, clarity, benefit, and completeness. However, GPT-4 scored higher in all criteria assessed including total mean grades (Table 2).

These findings are promising when considering the potential benefits of implementing an NLP model, like ChatGPT, into the field of medicine, and in agreement with this literature [10-15]. The visible advantages are manifold, with one of the standout features being the rapid retrieval of information and examination of the literature. ChatGPT’s strength lies in its capacity to quickly access a wide array of medical data from various sources. By offering doctors immediate entry to the newest findings, clinical standards, and specific cases, ChatGPT acts as a catalyst for keeping them aligned with the ever-changing medical landscape. This ability enhances physicians’ capacity to make educated judgments when dealing with intricate or uncommon medical scenarios.

Additionally, ChatGPT’s aptitude in understanding natural language equips it to thoroughly examine patient symptoms and medical backgrounds. This can lead to the suggestion of possible diagnoses, as well as offering alternative diagnoses for reflection. While it does not substitute for hands-on clinical expertise, ChatGPT proves to be an invaluable asset in assisting physicians to pinpoint diagnostic avenues and contemplate less evident conditions [16].

Furthermore, ChatGPT’s skill in scrutinizing medical publications and clinical studies empowers it to put forth suitable treatment recommendations rooted in the most up-to-date scientific proof. Doctors can leverage ChatGPT to weigh different treatment approaches, potential adverse effects, and counterindications, thus paving the way for customized and well-informed therapeutic choices [23].

The superior performance grades attributed to ChatGPT-4 align with expectations and are consistent with prior research comparing these 2 models within the health care domain [19,24-26]. This highlights the enhanced language model that ChatGPT-4 possesses, having benefitted from a broader and more varied data set during its development phase. Such improvements allow ChatGPT-4 to detect more complex linguistic patterns, thereby improving its ability to comprehend and generate contextually relevant responses. Furthermore, the inclusion of a more substantial corpus of medical literature, scientific articles, and ethical guidelines equips ChatGPT-4 with a wider base of knowledge. This rich repository of information empowers the model to provide more comprehensive and nuanced answers when faced with medical inquiries and ethical dilemmas.

Our study also explored an intriguing research question related to ChatGPT’s role in supporting decision-making in the nuanced area of ethical dilemmas. Since ethical considerations are a fundamental part of medical practice, they often create intricate scenarios for medical professionals. ChatGPT has the potential to act as a significant tool in this aspect, providing insights into ethical principles and the resolutions of past cases. While the final ethical determinations are the responsibility of the physician, ChatGPT’s assistance can guide them through multifaceted ethical conundrums, thereby enhancing the focus on patient-centered care. Hence, our data reveals that questions related to ethics consistently garnered the highest evaluations (as seen in Table 4 and Figures 1 and 2). These observations emphasize ChatGPT’s impressive ability to grapple with ethical dilemmas, regardless of their innate complexity. The model’s performance in this vital area is praiseworthy, even outperforming its evaluations in responding to information-based questions.

Interestingly, for both GPT-4 and GPT-3.5, senior physicians consistently rated the responses higher across all criteria. The variation was most notable in the assessment of the model’s benefits and completeness. These differences might be explained by the combination of higher experience with complexity, higher familiarity with research and guidelines, higher critical analysis skills, more experience navigating ethical dilemmas, and a broader interdisciplinary perspective possessed by senior physicians that likely contributes to their higher ratings of ChatGPT’s responses. However, further research is warranted to confirm our results.

### Limitations

Our study had several limitations. First, the expert panel that formulated the questions was composed of only 4 experts, while the group that assessed the questions included 8 physicians. Although the evaluating group was quite diverse, the findings might not accurately reflect the views of the broader physician community within these specialties. This small, potentially nonrepresentative sample could lead to biases in focus areas, subjective interpretations, and variability in expertise. Subjectivity in assessing responses, especially in relevance and clarity, combined with individual preconceptions about AI’s capabilities, might skew results. Expanding the expert pool and incorporating diverse perspectives from different subspecialties and health care settings, along with methodologies to adjust for individual biases, could mitigate these limitations in future studies.

Second, the assessment of ChatGPT’s efficacy relied on subjective judgments from 2 groups of physicians, which could introduce bias and inconsistency. Nonetheless, medical inquiries often encompass intricate matters that defy simple quantification, and the study intended to gauge its applicability to everyday medical work. Hence, personal assessment plays a vital role in this examination, ensuring that ChatGPT’s responses are pertinent, lucid, evidence-supported, legitimate, and worthwhile.

Third, this study concentrated solely on ChatGPT’s capability to respond to questions within specific subdisciplines of internal medicine, emergency medicine (ER), and ethics, leaving its potential in other medical fields unexplored. Additional studies are required to scrutinize ChatGPT’s performance across a broader spectrum of medical areas. Fourth, our examination was restricted to both versions of ChatGPT, raising the possibility that the findings might have differed with an alternate language model. Further research is needed to ascertain how applicable our conclusions might be to other large language models and varying contexts.

### Conclusion

In conclusion, the potential of ChatGPT in aiding physicians in addressing common medical problems is promising. As technology continues to advance, integrating ChatGPT into clinical workflows may become a valuable asset, enhancing diagnostic accuracy, treatment decisions, and ethical considerations. Nevertheless, it is essential to acknowledge that ChatGPT’s role should complement rather than replace clinical expertise and human judgment. As the technology evolves, further research and validation studies are warranted to optimize its abilities, ensuring safe and effective use in clinical settings.

## Appendix

Multimedia Appendix 1 Original Questions and Responses Generated by GPT-3.5 and GPT-4.

## Abbreviations

AI: artificial intelligence
ER: emergency room
NLP: natural language processing

## Data Availability

All data generated or analyzed during this study are included in this published article.
